# *Aintegumenta *and *Aintegumenta-Like6 *regulate auxin-mediated flower development in Arabidopsis

**DOI:** 10.1186/1756-0500-4-176

**Published:** 2011-06-07

**Authors:** Beth A Krizek

**Affiliations:** 1 Dept. of Biological Sciences, University of South Carolina, Columbia, SC, 29208, USA

## Abstract

**Background:**

Two related genes encoding AP2/ERF-type transcription factors, *AINTEGUMENTA *(*ANT*) and *AINTEGUMENTA-LIKE6 *(*AIL6*), are important regulators of floral growth and patterning in Arabidopsis. Evidence suggests that these genes promote several aspects of flower development in response to auxin. To investigate the interplay of *ANT*, *AIL6 *and auxin during floral development, I have examined the phenotypic consequences of disrupting polar auxin transport in *ant*, *ail6 *and *ant ail6 *mutants by either genetic or chemical means.

**Results:**

Plants containing mutations in *ANT *or *AIL6 *alone or in both genes together exhibit increased sensitivity to disruptions in polar auxin transport. Both genes promote shoot growth, floral meristem initiation and floral meristem patterning in combination with auxin transport. However, differences in the responses of *ant *and *ail6 *single mutants to perturbations in auxin transport suggest that these two genes also have non-overlapping activities in each of these developmental processes.

**Conclusions:**

The enhanced sensitivity of *ant *and *ail6 *mutants to alterations in polar auxin transport suggests that these mutants have defects in some aspect of auxin physiology. The inability of *ant ail6 *double mutants to initiate flowers in backgrounds disrupted for auxin transport confirm the proposed roles for these two genes in floral meristem initiation.

## Background

The phytohormone auxin is a central regulator of many aspects of plant growth and development. Within the shoot apical meristem, auxin accumulates in a graded and dynamic manner with sites of auxin maxima correlating with the sites of lateral organ initiation [1-3]. The formation of auxin gradients within the meristem appears to result primarily from directional transport of the hormone and local biosynthesis (reviewed in [4]). Directional transport is mediated by PINFORMED (PIN) proteins, which act as auxin efflux carriers and exhibit polarized plasma membrane localization [5]. Mutations in *PIN1 *result in inflorescences that do not initiate floral meristems and instead grow as pin-like structures [6]. This phenotype can be rescued by application of auxin to the meristem, demonstrating the importance of auxin to floral meristem initiation and the pivotal role that *PIN1 *plays in the generation of auxin gradients within the meristem [7]. These conclusions are also supported by studies showing that wild-type plants grown in the presence of the auxin transport inhibitor *N*-1-naphthylphthalamic acid (NPA) phenocopy *pin1 *mutants [6].

In addition to regulating floral meristem outgrowth, auxin also regulates floral organ development. Disruptions in polar auxin transport via transient NPA application result in flowers with reduced numbers of floral organs, narrower sepals and petals, malformed anthers and gynoecium with altered patterning [8]. These alterations in gynoecium patterning are very similar to those resulting from mutations in *ETTIN *(*ETT*), which encodes an auxin response factor (ARF) [8-10]. Mutations in *PIN1 *or *PINOID *(*PID*), which encodes a Ser-Thr kinase that controls PIN1 polarity, can produce several abnormal flowers prior to inflorescence termination in a pin-like structure [6,11-13]. These flowers exhibit a range of defects that can include alterations in organ number (typically fewer sepals and stamens and more petals), fusion of floral organs, and valveless gynoecia [6,13]. Disruptions in flower development have also been observed in plants defective in auxin biosynthesis. Biosynthesis of the major auxin, indole-3-acetic acid (IAA), involves both tryptophan (Trp)-dependent and Trp-independent pathways (reviewed in [14]). Mutations in multiple members of the *YUCCA *(*YUC*) family of flavin monooxygenases, which act in the tryptamine (TAM) Trp-dependent pathway, display reductions in floral organ number, altered organ morphology, valveless gynoecia and sterility [15]. Similar phenotypes are observed in plants mutant for tryptophan aminotransferase *TAA1 *and the related *TAR2 *gene, which act in the indole-3-pyruvic acid (IPA) Trp-dependent pathway [16,17].

The molecular mechanisms by which auxin regulates floral meristem outgrowth and organogenesis within the flower are not well defined. Real time imaging of inflorescence meristems has demonstrated that PIN1 upregulation in floral anlagen is correlated with downregulation of the meristem regulator SHOOTMERISTEMLESS (STM) as well as the boundary protein CUP-SHAPED COTYLEDON 2 (CUC2) [2]. Two genes that are likely to promote floral meristem initiation and specification downstream of auxin are the growth-promoting gene *ANT *and the floral meristem identity gene *LEAFY *(*LFY*). These two genes are early markers of a floral meristem fate and their expression is altered in *pin1 *mutants [18-20]. In addition, exposure of wild-type inflorescences to NPA results in a decrease in *ANT *mRNA accumulation in floral anlagen as early as 24 hours after treatment [21]. These data are consistent with a model in which auxin accumulation upregulates *ANT *and *LFY *expression to promote primordium outgrowth and floral identity, respectively [18]. However *ant *mutants do not display any defects in floral meristem initiation. Thus either *ANT *does not play a role in floral meristem initiation or it acts in parallel with other genes to promote primordium outgrowth.

Besides its proposed role in floral meristem initiation, *ANT *regulates floral meristem patterning, specification of floral organ identity, growth of floral organs and gynoecium patterning [19,21-25]. In two of these processes (lateral organ growth and gynoecium patterning) *ANT *function been linked directly to auxin [25,26]. *ANT *promotes growth downstream of the auxin-inducible gene *ARGOS *(auxin-regulated gene involved in organ size), while in maturing organs *ANT *expression is repressed by *ARF2*, a repressor of organ growth [26,27]. In the gynoecium, *ANT *acts together with *REVOLUTA *and polar auxin transport to specify development of the carpel medial domain [25].

In several of its roles in flower development, *ANT *acts in a partially redundant manner with the related *AINTEGUMENTA-LIKE6 *(*AIL6*). While *ail6 *flowers have a wild-type appearance, *ant ail6 *flowers have more severe defects than *ant. ant ail6 *flowers lack petals, stamens and normal gynoecium, are dramatically reduced in size, and exhibit defects in floral organ positioning [21]. Altered expression of the auxin-responsive reporter *AGH3-2:GUS *in *ant ail6 *inflorescence meristems and flowers suggests that these floral defects may be a consequence of altered patterns of auxin accumulation and/or responsiveness [21]. In addition to floral defects, *ant ail6 *plants exhibit decreased apical dominance, reduced stature, and altered vascular patterning, phenotypes similar to those found in plants disrupted in auxin physiology [21].

Like *ANT*, *AIL6 *expression is upregulated in incipient lateral organ primordia. This suggests that *AIL6 *might act redundantly with *ANT *in floral meristem initiation, but *ant ail6 *double mutants are still able to initiate floral meristems. *ant ail6 *inflorescence meristems do eventually stop initiating flowers, but this is due to a general growth arrest of the entire inflorescence apex [21] rather than specific termination of floral meristem initiation with continued growth of the inflorescence apex as is observed in *pin1 *mutants. Thus, it is still not known whether *ANT *and *AIL6 *regulate floral meristem initiation.

To further probe the roles of *ANT *and *AIL6 *in floral development processes potentially regulated by auxin, I have examined the consequences of losing *ANT *and *AIL6 *function singly or together in plants compromised for auxin transport via genetic or pharmacological means. These experiments demonstrate that both mutants are sensitized to defects in auxin transport, consistent with a role for these genes in regulation of some aspect of auxin physiology during flower development. The phenotypic consequences of loss of *ANT *function and loss of *AIL6 *function under the same conditions are somewhat different, suggesting that these two genes make distinct contributions to early stages of flower development. Floral meristem initiation is completely suppressed in plants compromised in polar auxin transport and containing mutations in both *ANT *and *AIL6*, demonstrating that these two genes do function in the initiation of floral meristems from the inflorescence meristem.

## Findings

### Mutations in *ANT *and *AIL6 *enhance the floral initiation and floral patterning defects of *pid *mutants

**Figure 1:**
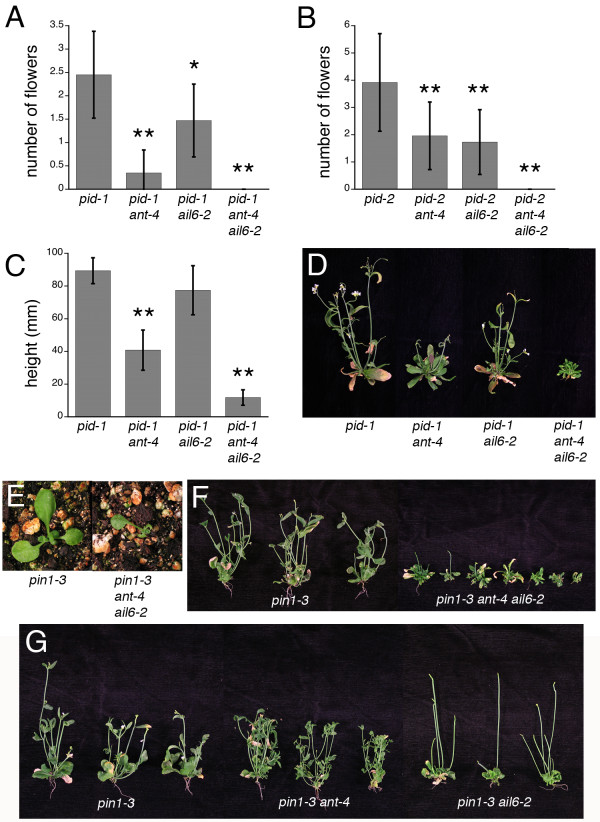
***ant-4 *and *ail6-2 *enhance the shoot and inflorescence defects of *pid *and *pin1 *mutants**. A. Number of flowers produced on *pid-1*, *pid-1 ant-4*, *pid-1 ail6-2 *and *pid-1 ant-4 ail6-2 *plants. B. Number of flowers produced on *pid-2*, *pid-2 ant-4*, *pid-2 ail6-2 *and *pid-2 ant-4 ail6-2 *plants. C. Height of *pid-1*, *pid-1 ant-4*, *pid-1 ail6-2 *and *pid-1 ant-4 ail6-2 *plants. Data in A-C are means ± SD. P values were determined by unpaired Student's t test (* P ≤ 0.01, ** P ≤ 0.0001). D. 39 day old *pid-1*, *pid-1 ant-4*, *pid-1 ail6-2 *and *pid-1 ant-4 ail6-2 *plants. E. 23 day old *pin1-3 *and *pin1-3 ant-4 ail6-2 *plants. F. 54 day old *pin1-3 *and *pin1-3 ant-4 ail6-2 *plants. G. 54 day old *pin1-3*, *pin1-3 ant-4 *and *pin1-3 ail6-2 *plants.

Because *pid *mutants make several flowers prior to termination of the inflorescence meristem, they provide a sensitized background in which to study the role of *ANT *and *AIL6 *in auxin-mediated floral meristem initiation. I introduced *ant-4*, *ail6-2 *and *ant-4 ail6-2 *into the strong *pid-1 *allele and the intermediate *pid-2 *allele [13]. No flowers were produced in *pid-1 ant-4 ail6-2 *or *pid-2 ant-4 ail6-2 *triple mutants (Figure 1A, B). While flowers were produced in *pid-1 ant-4*, *pid-1 ail6-2*, *pid-2 ant-4 *and *pid-2 ail6-2 *plants, they were reduced in number compared to *pid-1 *and *pid-2 *single mutants (Figure 1A, B). These results indicate that *ANT *and *AIL6 *have partially overlapping roles in promoting floral meristem initiation.

**Figure 2:**
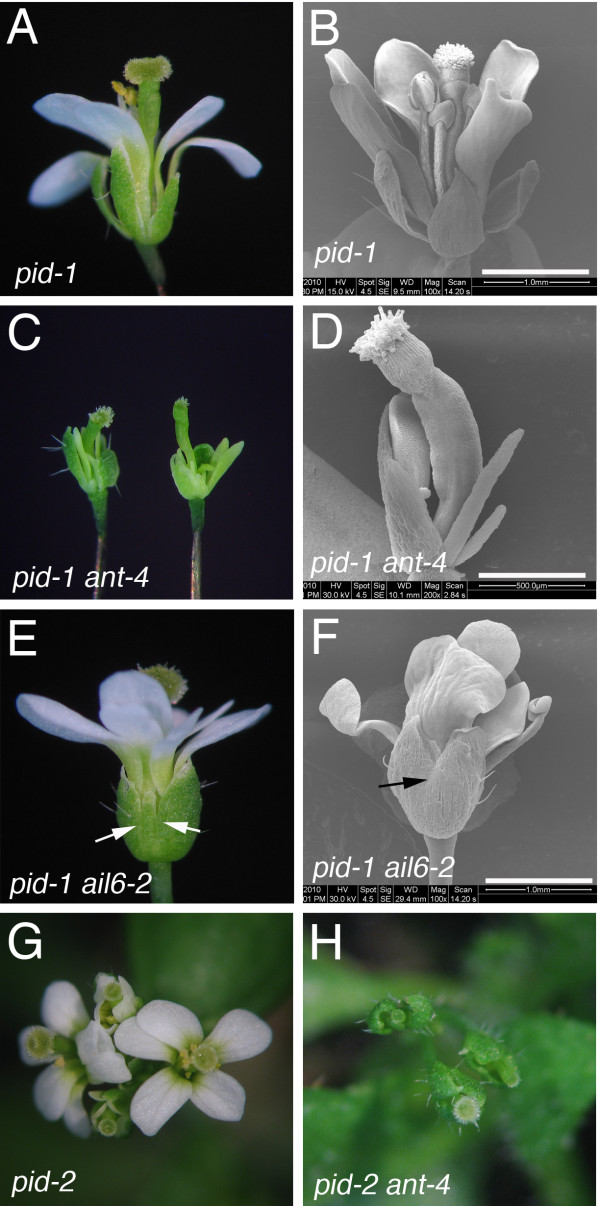
***ant-4 *and *ail6-2 *enhance the floral defects of *pid***. A. *pid-1 *flower. B. Scanning electron micrograph of a *pid-1 *flower. C. *pid-1 ant-4 *flowers. D. Scanning electron micrograph of a *pid-1ant-4 *flower. E. *pid-1 ail6-2 *flower. Arrows point to fused sepals. F. Scanning electron micrograph of a *pid-1 ail6-2 *flower. The arrow points to a region of sepal fusion. G. *pid-2 *inflorescence. H. *pid-2 ant-4 *inflorescence. Size bars correspond to 1 mm in B, F; 500 μM in D.

The flowers produced by *pid-1 ant-4 *and *pid-1 ail6-2 *plants exhibited more severe defects than those observed in *pid-1 *(Figure 2A-F). *pid-1 ant-4 *flowers were extremely small with reduced numbers of petals and stamens (Figure 2C, D). A similar phenotype was observed in *pid-2 ant-4 *flowers (Figure 2G, H). The dramatic effect on petal number was surprising since *ant-4 *flowers exhibit only slight reductions in petal number in early-arising flowers while *pid *flowers have increased numbers of petals [13,21]. *pid-1 ail6-2 *flowers resembled *pid-1 *flowers except for an increased incidence of sepal fusion. Eighty percent of *pid-1 ail6-*2 flowers exhibited sepal fusion while 41.2% of *pid-1 *flowers exhibited sepal fusion (Figure 2E, F). *ail6-2 *single mutants exhibited no sepal fusion or any other alterations in flower development compared with wild type [21].

### Mutations in *ANT *and *AIL6 *enhance shoot defects in plants genetically disrupted for auxin transport

The *pid-1 ant-4 ail6-2 *triple mutants also exhibited severe defects in leaf size and stem growth. Leaves of the triple mutant were thinner and shorter than in *pid-1 *(Figure 1D). The naked pin-like inflorescences of these plants were extremely short in height and often not visible above the basal rosette of the plant (Figure 1C, D). Leaf size and plant height were partially compromised in *pid-1 ant-4 *plants but not significantly altered in *pid-1 ail6-2 *plants (Figure 1C, D). Similar results were obtained with the *pid-2 *allele.

I also investigated the effect on introducing *ant-4 ail6-2 *into *pin *mutant backgrounds using *pin1-1 *(strong allele in Col) and *pin1-3 *(strong allele in L*er*). Similar to the results with *pid *mutants, *pin1-3 ant-3 ail6-2 *plants produce small leaves (Figure 1E) and short inflorescences (Figure 1F). Inflorescence height was somewhat variable in these plants (Figure 1F); in most cases the pin-like inflorescence did not extend beyond the basal rosette. Similar results were obtained with the *pin1-1 *allele. Interestingly, *pin1-3 ant-4 *and *pin1-3 ail6-2 *double mutants exhibited distinct phenotypes. *pin1-3 ant-4 *plants exhibited enhanced branching compared to *pin1-3*, while *pin1-3 ail6-2 *plants only rarely produced cauline leaves or axillary branches from the primary inflorescence (Figure 1G).

### *ant *mutants exhibit increased sensitivity to the effects of NPA on floral organ development

**Figure 3:**
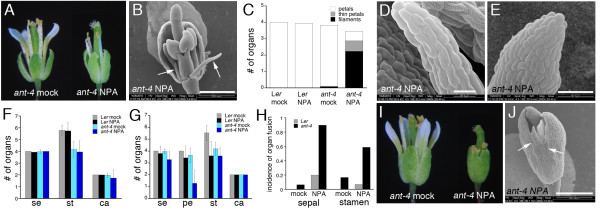
**Effects of NPA treatment on *ant-4 *flowers**. A. Mock (left) and NPA (right) treated *ant-4 *flowers 11 days post treatment. B. Scanning electron micrograph of a NPA treated *ant-4 *flower fixed 9 days post treatment. Arrows point to filaments present in the second whorl. C. Organ types found in second whorl of mock and NPA-treated L*er *and *ant-4 *flowers. D. Scanning electron micrograph of a second whorl filament. E. Scanning electron micrograph of a second whorl thin petal. F. Floral organ number in mock and NPA treated L*er *and *ant-4 *inflorescences from 10-12 days post treatment. Data are means ± SD. G. Floral organ number in mock and NPA treated L*er *and *ant-4 *inflorescences from 13-15 days post treatment. Data are means ± SD. H. Incidence of sepal fusion and stamen fusion in mock and NPA treated L*er *and *ant-4 *inflorescences. Sepal fusion was counted in flowers maturing at 13-15 days post treatment while stamen fusion was counted in flowers maturing 10-15 days post treatment. I. Mock (left) and NPA (right) treated *ant-4 *flowers 13 days post treatment. J. Scanning electron micrograph of an NPA-treated *ant-4 *flower that exhibits sepal fusion. Arrows point to fused sepals. Size bars correspond to 500 μM in B, J; 20 μM in D; 50 μM in E.

I next examined the consequence of inhibiting polar auxin treatment via application of 10 μM NPA to *ant-4 *flowers. This concentration of NPA had some phenotypic consequences on wild-type Arabidopsis flower development, such as reductions in stamen number (Figure 3G) but does not dramatically reduce floral meristem initiation, as is the case when wild-type inflorescences are treated with 100 μM NPA [8]. Thus it can be used to examine the relative sensitivity of *ant-4 *and L*er *flowers to NPA treatment. Examination of mock and NPA-treated *ant-4 *plants revealed temporally distinct phenotypes. The first effects of NPA treatment were observed in *ant-4 *flowers opening approximately 10 days after treatment and continued through day 12 after treatment. A second phenotypic class was observed in *ant-4 *flowers opening 13-15 days post treatment.

In 10-12 day post NPA-treated *ant-4 *flowers, second whorl petals were often replaced with filaments or very thin petals (Figure 3A-C). Organs were classified as filaments if they were radially symmetric and as thin petals if they had a flattened appearance. Alterations in petal development were not observed in 10-12 day post mock-treated *ant-4 *plants or NPA-treated L*er *plants (Figure 3A, C). To further characterize the second whorl organs that develop in these NPA-treated *ant-4 *flowers, SEM was used to examine their epidermal surface morphologies. Epidermal cells of the filaments lacked petal cell shape and cuticular thickenings suggesting that these organs did not possess any petal identity (Figure 3D). Epidermal cells of thin petals did show cuticular thickenings characteristic of petals (Figure 3E). However, these organs often lacked the conical cells normally present on the adaxial surface of petals, suggesting that these organs, like the filaments, had disruptions in organ polarity. No significant differences were observed in the numbers of sepals, stamens or carpels of 10-12 day post NPA-treated *ant-4 *or L*er *plants (Figure 3F). Occasionally, a valveless gynoecia phenotype was observed in the fourth whorl of 10-12 day post NPA-treated *ant-4 *flowers.

In 13-15 day post NPA-treated *ant-4 *plants, petals were often missing entirely (rather than being replaced with filaments) (Figure 3G) and sepals were often fused to each other (Figure 3H-J). The incidence of sepal fusion was much greater in NPA-treated *ant-4 *flowers (0.90 incidence/flower) compared with NPA-treated L*er *flowers (0.20 incidence/flower) (Figure 3H).

The primary effect of 10 μM NPA treatment on L*er *flowers was a decrease in stamen number (Figure 3G). Stamen number after NPA treatment was similar in L*er *and *ant-4 *flowers even though mock or untreated *ant-4 *flowers produced fewer stamens than L*er *(Figure 3G). In addition, the incidence of stamen fusion was increased in 10-15 day post NPA-treated *ant-4 *flowers (0.59 incidence/flower) compared to NPA-treated L*er *flowers (0.07 incidence/flower) (Figure 3H).

### *ail6 *mutants exhibit increased sensitivity to the effects of NPA on floral meristem initiation

**Figure 4:**
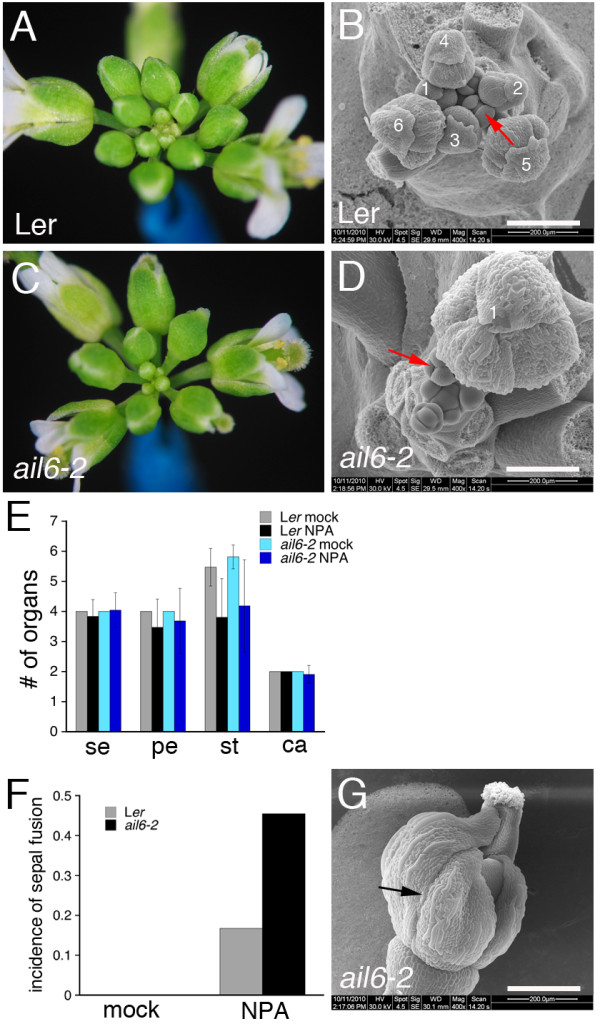
**Effects of NPA treatment on *ail6-2 *inflorescences**. A. L*er *inflorescence 11 days post NPA treatment. B. Scanning electron micrograph of L*er *inflorescence fixed 9 days post NPA treatment. The red arrow points to a stage 4 flower and the numbers mark all flowers initiated prior to the stage 4 flower. C. *ail6-2 *inflorescence 11 days post NPA treatment. D. Scanning electron micrograph of *ail6-2 *inflorescence fixed 9 days post NPA treatment. The red arrow points to a stage 4 flower. The number 1 marks the last flower initiated prior to temporary termination of floral meristem initiation. E. Floral organ number in mock and NPA treated L*er *and *ail6-2 *inflorescences from 12-15 days post treatment. Data are means ± SD. F. Incidence of sepal fusion in mock and NPA treated L*er *and *ail6-2 *flowers counted 12-15 days post treatment. G. Scanning electron micrograph of an NPA-treated *ail6-2 *flower that exhibits sepal fusion. Arrows point to a region of fusion. Size bars correspond to 200 μM in B, D; 200 μM in G.

Treatment of L*er *inflorescences with 10 μM NPA had no dramatic effects on floral meristem initiation (Figure 4A, B). However, treatment of *ail6-2 *inflorescences with 10 μM NPA resulted in a temporary suspension of floral meristem initiation (Figure 4C, D). Since primordia initiation was restarted at a later date, there was a gap in the developmental series of floral buds represented on an inflorescence (Figure 4C, D). SEM analyses suggested that this time period corresponds to the initiation of at least six flowers in wild type (Figure 4B, D). Floral organ number was similarly affected in NPA-treated L*er *and *ail6-2 *flowers (Figure 4E). While sepal number was similar in NPA-treated L*er *and *ail6-2 *flowers, the incidence of sepal fusion was increased in NPA-treated *ail6-2 *flowers (0.46 incidence/flower) compared to NPA-treated L*er *flowers (0.16 incidence/flower) (Figure 4F, G). No obvious effect on floral meristem initiation or floral organ development was observed in *ant-4 ail6-2 *plants treated with NPA as compared with mock-treated plants. Any effect on floral meristem initiation would be difficult to observe since termination of the inflorescence meristem occurs in untreated *ant-4 ail6-2 *inflorescences prior to the expected effect of NPA treatment.

## Discussion

### *ANT *and *AIL6 *promote floral meristem initiation

Although it has been proposed that *ANT *promotes primordium outgrowth in response to auxin accumulation, neither *ant *nor *ant ail6 *double mutants display defects in floral meristem initiation from the inflorescence meristem. The results presented here provide the first evidence that *ANT *and *AIL6 *are important factors regulating floral meristem initiation. Combining either *ant *or *ail6 *with *pid *reduces the number of flowers initiated by the inflorescence meristem, while the loss of both *ANT *and *AIL6 *function together prevents the formation of any flowers in *pid *mutants. These results suggest that *ANT *and *AIL6 *act in a redundant fashion to promote floral meristem initiation. Other pieces of evidence however suggest that the activities of these two genes are not completely overlapping and that *AIL6 *may play a more important role in this process as compared with *ANT*. Floral meristem initiation was temporarily terminated in *ail6-2 *inflorescences treated with 10 μM NPA but not in L*er *or *ant-4 *inflorescences similarly treated. Thus floral meristem initiation is inhibited by lower levels of NPA in *ail6-2 *inflorescences. Furthermore, loss of *AIL6 *function in the *pin1-*3 background results in a nearly complete loss of lateral branches from inflorescences.

### *ANT *and *AIL6 *act with polar auxin transport in floral meristem patterning

One defect in flower development observed in mutants with disruptions in polar auxin transport such as *pid *[13] or in wild-type plants treated with NPA was fusion of floral organs. This may be a consequence of alterations in the positioning or outgrowth of organ primordia from the floral meristem and/or the inability to establish boundaries between adjacent organs. It is known that polar auxin transport is critical to cotyledon boundary formation during embryogenesis [28]. In postembryonic development, separation between lateral organs and the meristems from which they arise is also likely to involve polar auxin transport (reviewed in [4,29]). Live imaging of inflorescence meristem shows that upregulation of PIN1 expression in lateral organ founder cells was correlated with reduced expression of meristem and boundary markers such as STM and CUC2 [2]. Correspondingly, boundary genes such as *JAGGED LATERAL ORGAN *(*JLO*) appear to repress *PIN1 *and promote *STM *expression in boundary cells [30]. These antagonistic relationships result in the separation of indeterminate cells within the meristem and determinate cells within the lateral organ primordia [29]. The results presented here showing floral organ fusion in some NPA treated wild-type flowers suggest that a common mechanisms is involved in organ separation in shoot apical meristems and floral meristems. Sepal fusion was enhanced in *pid-1 ail6-2 *flowers compared to *pid-1 *flowers and in NPA treated *ant-4 *and *ail6-2 *flowers as compared with NPA treated L*er *flowers. Thus, *ANT *and *AIL6 *appear to function together with polar auxin transport in separation of sepals, while *ANT *also acts in stamen boundary specification. These data provide a link between auxin and the role of *ANT *and *AIL6 *in floral meristem patterning.

### *ANT *promotes petal development in combination with auxin transport

*ant-4 *mutants in which polar auxin transport was reduced also exhibited severe effects on petal development. Few petals were initiated in *pid ant-4 *double mutants or in the later-arising NPA-treated *ant-4 *flowers. The distinct temporal effects observed after NPA treatment on *ant-4 *flowers were likely a consequence of the floral development stage at which auxin transport was inhibited. Flowers maturing 10-12 days after NPA treatment presumably correspond to those in which sepal and petal primordia were already initiated while flowers maturing 13-15 days after NPA treatment most likely correspond to younger floral meristems in which sepal and petal primordia had not yet initiated. In L*er *plants, the length of time between floral meristem initiation and flower opening is approximately 13.25 days [31]. In the earliest flowers affected by NPA treatment, second whorl organs were present but often developed as filaments or very thin petals. The lack of petal blade outgrowth and the absence of conical cells in thin petals indicate that petal polarity is disrupted. A role for auxin in mediating adaxial/abaxial patterning during organogenesis has been suggested by the phenotype of plants lacking two related ARFs, ETT and ARF4 [32]. *ett arf4 *double mutants have defects in lateral organ polarity that mimic loss of function mutations in *KANADI *genes that specify abaxial identity [32]. In later-arising NPA-treated *ant-4 *flowers, second whorl organs were usually missing suggesting that polar auxin transport is required for petal initiation. It is not clear why petal initiation is particularly sensitive to NPA treatment in the *ant-4 *background.

## Conclusions

Here I show that combining mutations in *ANT *and *AIL6 *with disruptions in polar auxin transport results in severe effects on shoot and flower development. *ANT *and *AIL6 *appear to have both overlapping and distinct roles in the process of floral meristem initiation downstream of auxin, while *ANT *appears to play a more important role than *AIL6 *in later stages of auxin-mediated floral meristem patterning and floral organogenesis. The results are consistent with a model in which *ant *and *ail6 *mutants are disrupted in some aspect of auxin physiology. Furthermore they support the idea that all of the different functions of these two genes are linked with auxin dynamics within shoot tissues.

## Methods

### Plant materials and growth conditions

*Arabidopsis thaliana *ecotype Landsberg *erecta *(L*er*) was used as the wild type. Other mutants used in the study were *pid-1 *[13], *pid-2 *[13], *pin1-1 *[6], *pin1-3 *[13], *ant-4 *[33] and *ant-4 ail6-2 *[21]. Plants were grown on a soil mixture of Metro-Mix 360:perlite:vermiculite (5:1:1) under continuous light or in 16 hour days (100-150 μmol/m^2^/s) at a temperature of 22°C.

### Genetics and PCR genotyping

*ant-4 *and *ant-4/+ ail6-2 *plants were crossed to *pid-1/+*, *pid-2*, *pin1-1/+ *and *pin1-3/+ *plants. Double and triple mutants were identified in the F2 or later generations as plants with new phenotypes and confirmed by PCR genotyping. *ant-4 *and *ail6-2 *were PCR genotyped as described previously [21]. *pin1-3 *was PCR genotyped using PIN1-5: 5'-caccgctacgaacgatcatcaa-3' and PIN1-6: 5'-atgctttctgctgtgaagccag-3'. Digestion of the PCR product with ScaI produced two fragments of 449 bp and 529 bp for wild type and an intact 978 bp fragment for *pin1-3*.

### SEM

Tissue for SEM was fixed, dried, dissected and coated as described previously [23]. SEM analyses were performed on a FEI Quanta 200 ESEM.

### NPA treatment

L*er*, *ant-4, ail6-2 *and *ant-4 ail6-2 *inflorescences were painted with a 10 μM NPA (in 0.1% DMSO, 0.01% Silwet L-77) solution or a mock (0.1% DMSO, 0.01% Silwet L-77) solution twice (at time zero and 7 hours).

## Competing interests

The author declares that they have no competing interests.

## Authors' contributions

BAK is solely responsible for this work.
